# Distribution of polycyclic aromatic hydrocarbons in subcellular root tissues of ryegrass (*Lolium multiflorum *Lam.)

**DOI:** 10.1186/1471-2229-10-210

**Published:** 2010-09-22

**Authors:** Fuxing Kang, Dongsheng Chen, Yanzheng Gao, Yi Zhang

**Affiliations:** 1 Institute of Organic Contaminant Control and Soil Remediation, College of Resource and Environment Science, Nanjing Agricultural University, Nanjing 210095, China

## Abstract

**Background:**

Because of the increasing quantity and high toxicity to humans of polycyclic aromatic hydrocarbons (PAHs) in the environment, several bioremediation mechanisms and protocols have been investigated to restore PAH-contaminated sites. The transport of organic contaminants among plant cells via tissues and their partition in roots, stalks, and leaves resulting from transpiration and lipid content have been extensively investigated. However, information about PAH distributions in intracellular tissues is lacking, thus limiting the further development of a mechanism-based phytoremediation strategy to improve treatment efficiency.

**Results:**

Pyrene exhibited higher uptake and was more recalcitrant to metabolism in ryegrass roots than was phenanthrene. The kinetic processes of uptake from ryegrass culture medium revealed that these two PAHs were first adsorbed onto root cell walls, and they then penetrated cell membranes and were distributed in intracellular organelle fractions. At the beginning of uptake (< 50 h), adsorption to cell walls dominated the subcellular partitioning of the PAHs. After 96 h of uptake, the subcellular partition of PAHs approached a stable state in the plant water system, with the proportion of PAH distributed in subcellular fractions being controlled by the lipid contents of each component. Phenanthrene and pyrene primarily accumulated in plant root cell walls and organelles, with about 45% of PAHs in each of these two fractions, and the remainder was retained in the dissolved fraction of the cells. Because of its higher lipophilicity, pyrene displayed greater accumulation factors in subcellular walls and organelle fractions than did phenanthrene.

**Conclusions:**

Transpiration and the lipid content of root cell fractions are the main drivers of the subcellular partition of PAHs in roots. Initially, PAHs adsorb to plant cell walls, and they then gradually diffuse into subcellular fractions of tissues. The lipid content of intracellular components determines the accumulation of lipophilic compounds, and the diffusion rate is related to the concentration gradient established between cell walls and cell organelles. Our results offer insights into the transport mechanisms of PAHs in ryegrass roots and their diffusion in root cells.

## Background

Polycyclic aromatic hydrocarbons (PAHs) are a group of persistent organic contaminants (POPs) that are ubiquitous in the environment [1-3]. Their toxicity (e.g., mutagenic, carcinogenic) and potential of accumulation in biota have led to concern about their fate and transport in the environment [4-6]. The major sources of PAHs in the environment include incomplete combustion of organic residues (polymerization of benzene rings at high temperature), petroleum production, volcanic eruptions, and enzymatic polymerization of the benzene ring from plant exudates to the soil [7,8]. Although these contaminants are mainly metabolized and decomposed via environmental biotic and abiotic processes [9,10], PAHs in the environment have gradually increased over the past several decades. For example, in Daya Bay, South China, before 1955, the temporal distribution of PAH concentrations in sediments was below 150 μg·kg^-1 ^(dry weight), but by 1995, concentrations had risen to 300 μg·kg^-1 ^[11]. This increased PAH accumulation in the environment is because the rate of PAH release from anthropogenic activities is greater than the rate of natural attenuation.

Several remediation technologies and protocols have been developed to restore PAH-contaminated sites [7]. Phytoremediation is a potent and efficient approach that removes PAHs from contaminated sites into plants and decomposes them to less hazardous or non-hazardous forms with minimum input of chemicals and energy [7,12-15]. Previous studies have shown the efficacy of plant uptake and metabolism of PAHs in removing PAHs from the environment [16-18]. In general, two primary processes are responsible for PAH transfer and distribution in plant tissues: (1) transfer between plant tissues and cells driven by transpiration and the PAH concentration gradient across plant-cell components and (2) accumulation of PAHs in plant tissues, with the extent related to plant lipid contents [18-21]. However, the factors that influence PAH transfer and distribution in plants as well as their metabolism in cells are not clear. Plant uptake of PAHs from contaminated media is primarily through the roots and secondarily through leaves [16-18]. PAHs and their degradation products have frequently been detected within plant tissues [13]. A recent study has shown that in *Zea mays *phenanthrene can be metabolized into more polar products [22]. In another study, anthracene and formed metabolites were bound to several cell-wall components, such as pectin, lignin, hemicellulose, and cellulose [23]. Similarly, Wild et al. (2005) investigated the distributions of anthracene and its metabolites in *Zea mays *and suggested that the metabolism of anthracene occurs predominantly in the cell wall [24].

Uptake from water and soil via plant roots is a major pathway of PAH entry into plants. Wild et al. (2005) reported that PAHs first adsorbed to root surfaces and then passed through the membranes of adjoining cells before accumulating in cell walls and vacuoles [24]. The amount of uptake depended primarily on the lipid content of plant roots, in which protein, fats, nucleic acids, cellulose tissues, and other components all contain lipophilic components, which appear to be the primary domains where PAHs accumulate once they penetrate plant root cells [18]. Unfortunately, despite extensive studies on the transport of organic contaminants (especially PAHs) in plants, information about PAH distributions in intracellular tissues of plant roots, stalks, and leaves is lacking. This limits the development of mechanism-based phytoremediation strategies to better improve treatment efficiency.

In this study, we investigated the uptake and subcellular distributions of PAHs in root cells of ryegrass (*Lolium multiflorum *Lam.), which is widely used in the phytoremediation of PAH-contaminated sites owing to its fibrous root system and large specific root surface area. Our results will enhance the understanding of PAH transfer mechanisms in plants and their effects on the distribution of PAHs in plants.

## Results and Discussion

### Uptake of phenanthrene and pyrene by roots

**Figure 1:**
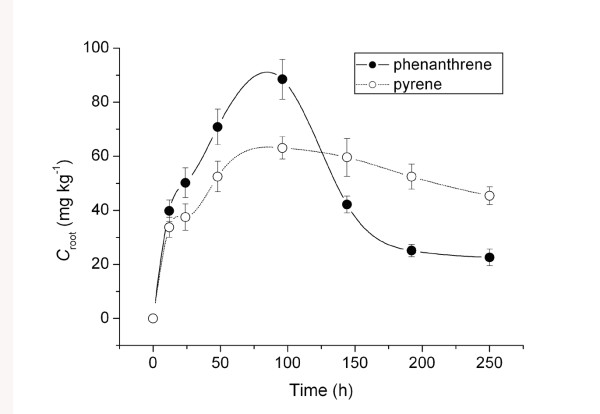
**Concentrations of phenanthrene and pyrene in ryegrass roots as a function of uptake time**. *C*_root _means concentrations of phenanthrene and pyrene in ryegrass root.

In Figure 1, the uptake of phenanthrene and pyrene from Hoagland medium by ryegrass roots is shown as a function of exposure time. The uptake rate and magnitude of uptake of phenanthrene and pyrene by ryegrass roots differed. Concentrations of phenanthrene and pyrene in roots increased with exposure time, reaching a maximum at ~100 h. Although phenanthrene concentrations in roots were higher within this timeframe, they were less than two times the concentration of pyrene, most likely because of a higher initial concentration in the medium of phenanthrene (2.5 mg·L^-1^) than of pyrene (0.5 mg·L^-1^). From 100 to 240 h, the phenanthrene concentration in roots decreased sharply from 90 to 18 mg·kg^-1^, whereas that of pyrene declined gradually from 60 to 48 mg·kg^-1^. These differing uptake patterns could result from a difference in the migration of phenanthrene and pyrene to ryegrass shoots, their degradation in roots, or both. The slower rate of pyrene reduction after 100 h indicates that pyrene is recalcitrant to metabolism in roots and that, as the more lipophilic compound, it exhibits a strong affinity for plant tissues, slowing its transport from roots to shoots.

**Figure 2:**
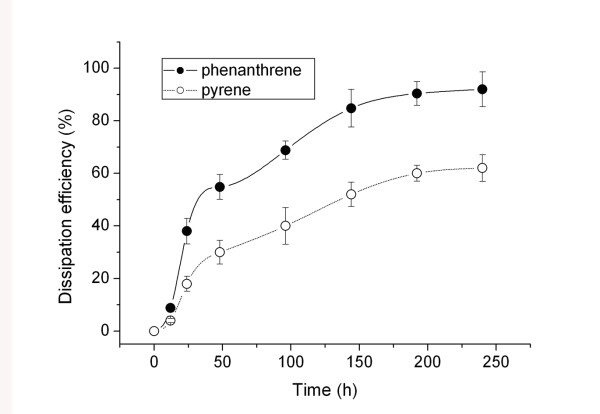
**Dissipation efficiency (%) of phenanthrene and pyrene by ryegrass from aqueous solution as a function of exposure time**. The values were defined as ratio of PAH removal to the initial concentration from the aqueous solution. It explained in principle the plant-affected dissipation of phenanthrene and pyrene from solution, and such dissipation was primarily related to plant accumulation and metabolism.

Although spontaneous volatilization could be a cause of PAH dissipation from water, it is thought to be primarily related to plant accumulation and metabolism [2]. Figure 2 shows the dissipation efficiency of PAHs from Hoagland solution by ryegrass, which we defined as the ratio of PAH removal to the initial concentration of PAH in aqueous solution. Dissipation efficiency increased gradually with exposure time, reaching 92% for phenanthrene and 62% for pyrene at 250 hours. This result is consistent with the relatively rapid reduction of phenanthrene in ryegrass roots, which could be due to its high metabolism rate in ryegrass, relatively quick migration from roots to shoots, or both.

**Figure 3:**
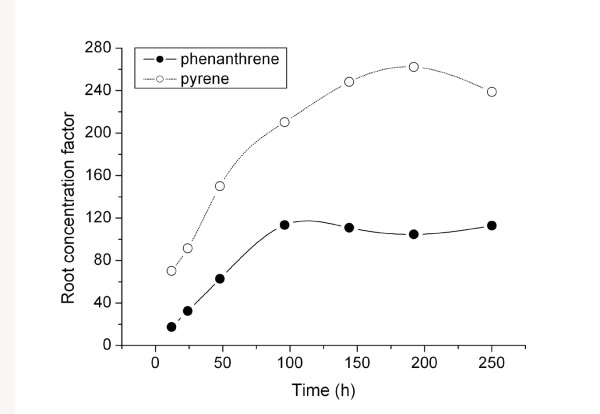
**Root concentration factor (RCF) of phenanthrene and pyrene for ryegrass uptake from aqueous solution**. Root concentration factor (RCF) describes the capability of roots to accumulate contaminants from the direct contact with the aqueous environment, which is here defined as the ratio of PAH concentration in root (*C*_root_) to that in culture medium (*C*_solution_), i.e., RCF = *C*_root_/*C*_solution_.

The root concentration factor (RCF) describes the capability of roots to accumulate contaminants from direct contact with an aqueous environment, which is here defined as the ratio of PAH concentration in roots (*C*_root_) to that in the culture medium (*C*_solution_): RCF = *C*_root_/*C*_solution _[25-27]. RCF values increased with increasing root/solution contact time before approaching a nearly constant value after 150 h (Figure 3). The RCF values of pyrene were about two times greater than those of phenanthrene. Our previous study indicated that the lipophilicity (e.g., log *K*_ow_) of a compound is a determinant of the magnitude of plant uptake [18]. The higher pyrene RCF is due to its greater lipophilicity (log *K*_ow _= 5.32) compared with that of phenanthrene (log *K*_ow _= 4.46). Gao et al. (2004) reported that pyrene uptake by plants from soil was 4-7 times greater than uptake of phenanthrene [18]. Together, these results suggest that more lipophilic organic contaminants have a higher propensity for uptake in plants via roots.

### Subcellular movement and distribution of phenanthrene and pyrene in root cells

**Figure 4:**
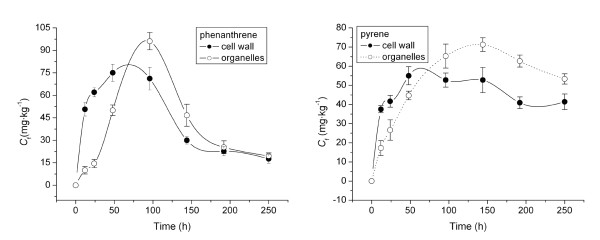
**Concentrations of phenanthrene (a) and pyrene (b) in root cell walls and organelles as a function of time**. *C*_f _means concentrations of phenanthrene and pyrene in root subcellular fractions.

Figure 4 shows phenanthrene and pyrene concentrations in root subcellular fractions. Phenanthrene concentrations in cell walls and organelles increased gradually to 79 and 95 mg·kg^-1^, respectively, and then decreased to 16.5 and 17 mg·kg^-1 ^as exposure time lengthened (Figure 4a). Pyrene underwent a similar uptake pattern: pyrene concentrations in cell walls and organelles rose to 58 and 71 mg·kg^-1^, respectively, and then decreased to 38 and 56 mg·kg^-1 ^(Figure 4b). Before 70 h of uptake, concentrations of both PAHs in cell walls were greater than those in organelles. Moreover, the uptake by organelle components was slower in reaching a maximum relative to cell walls. The two PAHs first adsorbed onto cell walls from the culture medium, and they then diffused into cell organelle components. After 70 h, a relatively higher concentration of both PAHs was found in the organelle fraction than in cell walls due to the greater accumulation of lipophilic compounds in the fraction containing a higher lipid content, i.e., organelle components. After 96 h of exposure, phenanthrene uptake rapidly decreased in root cell walls and organelles, whereas the decrease in pyrene was much slower in these two subcellular fractions. This trend is consistent with the uptake patterns of the two contaminants by ryegrass roots shown in Figure 1.

**Figure 5:**
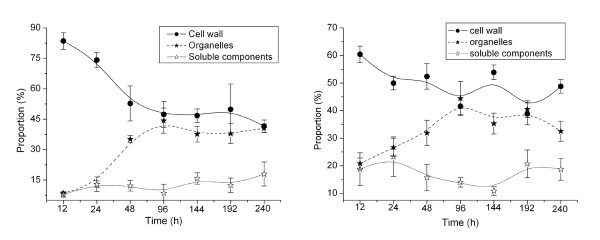
**Proportions of phenanthrene (a) and pyrene (b) distributed in cell water soluble fraction, wall and organelle as a function of uptake time**. The proportion of PAHs was calculated from the measurement of PAH in each fraction to the total amount in ryegrass root cells.

PAHs in roots were distributed into three subcellular fractions: water-soluble, cell wall, and organelle. The proportions of phenanthrene and pyrene in the water-soluble, wall, and organelle fractions are plotted against exposure time in Figure 5. From 12 h to 240 h of exposure, the percentage of phenanthrene in cell walls notably descended from 84% to 42% and pyrene decreased from 60% to 41%. Within the same period, phenanthrene and pyrene distributions in organelles increased from 8.5% to 41% and from 21% to 33%, respectively. Both PAHs showed a relatively small variation in the proportion in the aqueous soluble fraction, ~10-15% for phenanthrene and ~10-20% for pyrene. Thus, at the beginning of uptake (i.e., < 96 h), the decrease of PAH in cell walls largely corresponded to the increase in cell organelles, suggesting that PAHs first accumulated in cell walls via direct contact with Hoagland solution and then gradually transferred to fractions inside cells, such as organelles. After 96 h of exposure, the distributions of both PAHs in cell components approached a relatively stable state in cell walls and organelles (Figure 5). In ryegrass root cells, content in cell walls was 8.9%, and that in organelles was 6.0%. The relatively smaller organelle fraction accumulated a similar amount of each PAH compared with that in cell walls, likely owing to the higher lipid content of organelles. Generally, the uptake capability of root tissues for organic lipophilic compounds increases with *K*_ow _value (i.e., *K*_ow _> ~10^4^), with more lipophilic compounds showing a higher accumulation in plants, particularly in plant tissues containing a high lipid content [20,21,28,29]. Lipids in plant cell walls are composed mostly of polysaccharides (90%), with a few structural proteins, lignin, lectin, and mineral elements as well as a very small lipid component. In contrast, the lipid content of plant organelles is 15-30% [30], enabling them to draw PAHs from the cell wall. Thus, the relatively higher lipid content of the organelle fraction is believed to be responsible for the greater accumulation of PAHs, and the corresponding concentration gradient established between organelles and cell walls in the beginning stage of uptake (< 96 h) is the driving force for the diffusion of PAHs to interior cell components.

As shown in Figure 5, PAH concentrations in soluble components stayed nearly constant. The separated soluble cellular components, mainly consisting of cell solution and largely concentrated in the cell matrix between cells or organelles, can be regarded as an intracellular buffering distribution phase. Due to the hydrophobicity of PAHs, these aqueous substances were not easily enriched. Thus, the non-affinity between PAHs and soluble cellular components may result in distributive constant and low partitioning proportions.

**Figure 6:**
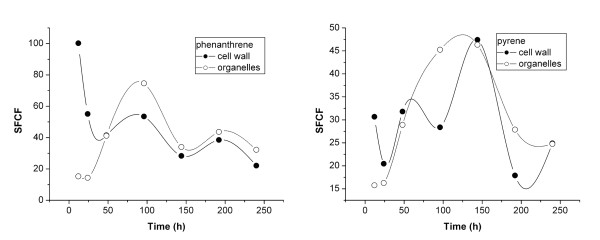
**Subcellular fraction concentration factors (SFCF) of phenanthrene (a) and pyrene (b) for ryegrass root uptake as a function of time 0~240 h**. SFCF was defined as the ratio of PAH concentration in subcellular fractions including cell wall and organelle to that in cell water-soluble components.

Subcellular fraction-concentration factor (SFCF) values, defined as the ratio of PAH concentration in subcellular fractions to that in water-soluble cellular components, are shown in Figure 6. The SFCF of phenanthrene in cell walls decreased from 101 to 20 L·kg^-1 ^over 240 h of exposure. For pyrene, the SFCF in cell walls first increased from 16 to 48 L·kg^-1 ^and then decreased rapidly to < 25 L·kg^-1^. The difference in cell-wall SFCFs of phenanthrene and pyrene likely resulted from the different properties of the two PAHs, as in the beginning stage, pyrene tended to accumulate more in cell walls than in water-soluble components owing to its higher log*K*_ow_.

In the initial 48 h, the SFCFs of the two PAHs were greater in cell walls than in cell organelles. After that period, organelle fraction SFCFs slightly exceeded those of cell walls. These results suggest that within the first 48 h of exposure, subcellular transport of PAHs occurred from cell walls to intracellular organelles as a result of the concentration gradient.

## Conclusions

Transpiration is generally considered to be the main transfer mechanism of PAHs in plants, such as from roots to stalks and leaves [9]. PAHs initially adsorb to plant cell walls and then gradually diffuse into subcellular tissues. The lipid contents of intracellular components determine the extent of lipophilic compound accumulation, and the diffusion rate is related to the concentration gradient established between cell walls and organelles inside cells. In addition, although both phenanthrene and pyrene are grouped among organic compounds that share similar properties, pyrene displays greater accumulation factors in subcellular walls and organelle fractions due to a higher log*K*_ow_. Our results will be useful in evaluating human exposure risks of PAH-contaminated crops and in developing appropriate strategies for the phytoremediation of PAH-contaminated sites.

### Remaining question

To our best knowledge, this is the first paper reporting the distribution of persistent organic pollutants (POPs), with PAHs as representatives, in plant subcellular tissues. It is noteworthy that in this study, although the negligible amounts of pure cell membrane could not be separated from other cell fractions by the centrifugal method and it was merged into the soluble components in the investigation, results of this work open new insights into POP subcellular transport and distribution in plants.

## Methods

### Chemicals

Phenanthrene and pyrene (> 98% purity) were purchased from Sigma-Aldrich GmbH (Munich, Germany). Table 1 lists some physicochemical properties of the two PAHs. Milli-Q grade water (Millipore, Billerica, MA, USA; 18.2 MΩ·cm^-1 ^resitivity) was used to prepare solutions. Hoagland solution was prepared in 1.0 L water as follows: KNO_3 _(607 mg), Ca(NO_3_)_2 _(945 mg), (NH_4_)_3_PO_3 _(115 mg), MgSO_4 _(493 mg), FeSO_4_·7H_2_O (13.9 mg), EDTA·2Na (18.65 mg), KI (0.00415 mg), H_3_BO_3 _(0.031 mg), MnSO_4 _(0.1115 mg), ZnSO_4 _(0.043 mg), Na_2_MoO_4 _(0.00125 mg), CuSO_4 _(0.000125 mg), and CoCl_2 _(0.000125 mg).

**Table 1:** Some physicochemical properties of phenanthrene and pyrene

PAHs	**Molar weight (g·mol**^**-1**^)	**Water solubility (mg·L**^**-1 **^**at 25°C)**	**log*K***_**ow**_
Phenanthrene	178.23	1.18	4.46
Pyrene	202.26	0.12	5.32

### Plant uptake experiment

Ryegrass root uptake of phenanthrene and pyrene from aqueous Hoagland solution was investigated using batch settings. A stock methanol solution of phenanthrene and pyrene was added to aqueous Hoagland solution according to the method described by Chapin et al. [31]. The methanol concentration in the Hoagland solution was less than 0.1% (vol/vol). Following germination in vermiculite, seedlings were transferred to a tray containing Hoagland solution and grown in a greenhouse at 25-30°C in daytime and 20-25°C at night. After about 2 weeks of growth, the plants were approximately 10 cm tall with relatively mature roots, and they were used for uptake experiments.

The seedlings were then transplanted to Hoagland culture solutions containing phenanthrene and pyrene. Twelve seedlings were cultured in each glass beaker, with roots submerged in the culture medium. Four replicates of each treatment were conducted. During the experimental period, the seedlings were incubated at 25-30°C during daytime and 20-25°C at night. The culture beakers were wrapped with black cloth to reduce the impact of potential photolysis. Each day, PAH-free control Hoagland solution was added to both the experimental and PAH-free control beakers to maintain the same initial volume of each treatment. At 12-, 24-, 48-, 96-, 168-, and 240-h exposure, the Hoagland solution and corresponding seedlings were sampled and prepared for PAH analysis. Seedling roots were washed several times using Milli-Q water and then separated into cell-wall and organelle fractions to measure PAH distributions at the subcellular level (see below).

### Subcellular fraction

A modification of the methods of Lai et al. (2006), Li et al. (2006), and Wei et al. (2005) was used to obtain subcellular fractions of root cells [32-34]. Briefly, fresh roots were mixed with extraction buffer containing 50 mM HEPES, 500 mM sucrose, 1.0 mM DTT, 5.0 mM ascorbic acid, and 1.0% (w/v) polyclar AT PVPP. The buffer solution pH was adjusted to 7.5 using 1.0 M NaOH. The root tissue extract was ground, passed through a 60-μm sieve, and subsequently centrifuged at 500 g for 5 min to obtain a pellet of cell debris. This pellet was referred to as the wall fraction of the root cells. The supernatant was then centrifuged at 10,000 g for 30 min to obtain the cell organelle fraction. All extraction steps were performed at 4°C. The dried cell-wall and organelle powders were placed in a vacuum freeze drier (Labconco, Kansas City, MO, USA) at -65°C. Fraction contents, determined gravimetrically, were 6.0% organelles and 8.9% cell walls, with the remainder being water and water-soluble fractions.

### Analysis of PAHs

PAHs were extracted from cell fractions using ultrasonication [18]. In brief, the cell fractions (cell walls and organelles) were freeze-dried and then extracted for 1 h, using an acetone and hexane mixture (vol/vol = 1:1), followed by 1 h of ultrasonic extraction. This acetone/hexane extraction step was repeated three times, and the collected extracts were combined. The solvents were then evaporated using a rotary evaporator and exchanged to 2 mL hexane, followed by a clean-up procedure through a 2-g silica gel column using an 11-mL 1:1 (v/v) elution of hexane and dichloromethane. The samples were then evaporated and exchanged to methanol, with a final volume of 2 mL. Phenanthrene and pyrene were analyzed using high-performance liquid chromatograph (LC-20AT; Shimadzu, Kyoto, Japan) equipped with a UV detector and a Ф4.6 × 150-mm reverse phase C_18 _column. The UV-detector wavelength was set at 254 nm. The mobile phase was spectrum-pure methanol with a flow rate at 1.0 mL·min^-1^, and the column temperature was 30°C.

### Statistical Analysis

All data were calculated using Origin version 7.0. Every data point in the Figures is an average value. The standard deviation (SD), obtained from four parallel samples using the Origin software, is shown in the Figures as an error bar. Data were analyzed using analysis of variance (ANOVA). The statistical package used was SPSS (Version 11.0), and the confidence limit was 95%.

## Authors' contributions

YZG performed the project planning. DSC performed the experimental work, while FXK, DSC, YZG, and YZ performed the data analysis. FXK, DSC, and YZG wrote the manuscript. All authors read and approved the final manuscript for submission.
